# Supplementary material for: Reasons for Initiation and Regular Use of Heated Tobacco Products among Current and Former Smokers in South Korea: Findings from the 2020 ITC Korea Survey
Source: Int J Environ Res Public Health. 2023 Mar 11;20(6):4963. doi: 10.3390/ijerph20064963 (PMC10048802; doi:10.3390/ijerph20064963)
Supplement: Supplementary file 1 [file ijerph-20-04963-s001.zip › ijerph-2134965-supplementary.pdf]

Figure S1. Study Flow Diagram

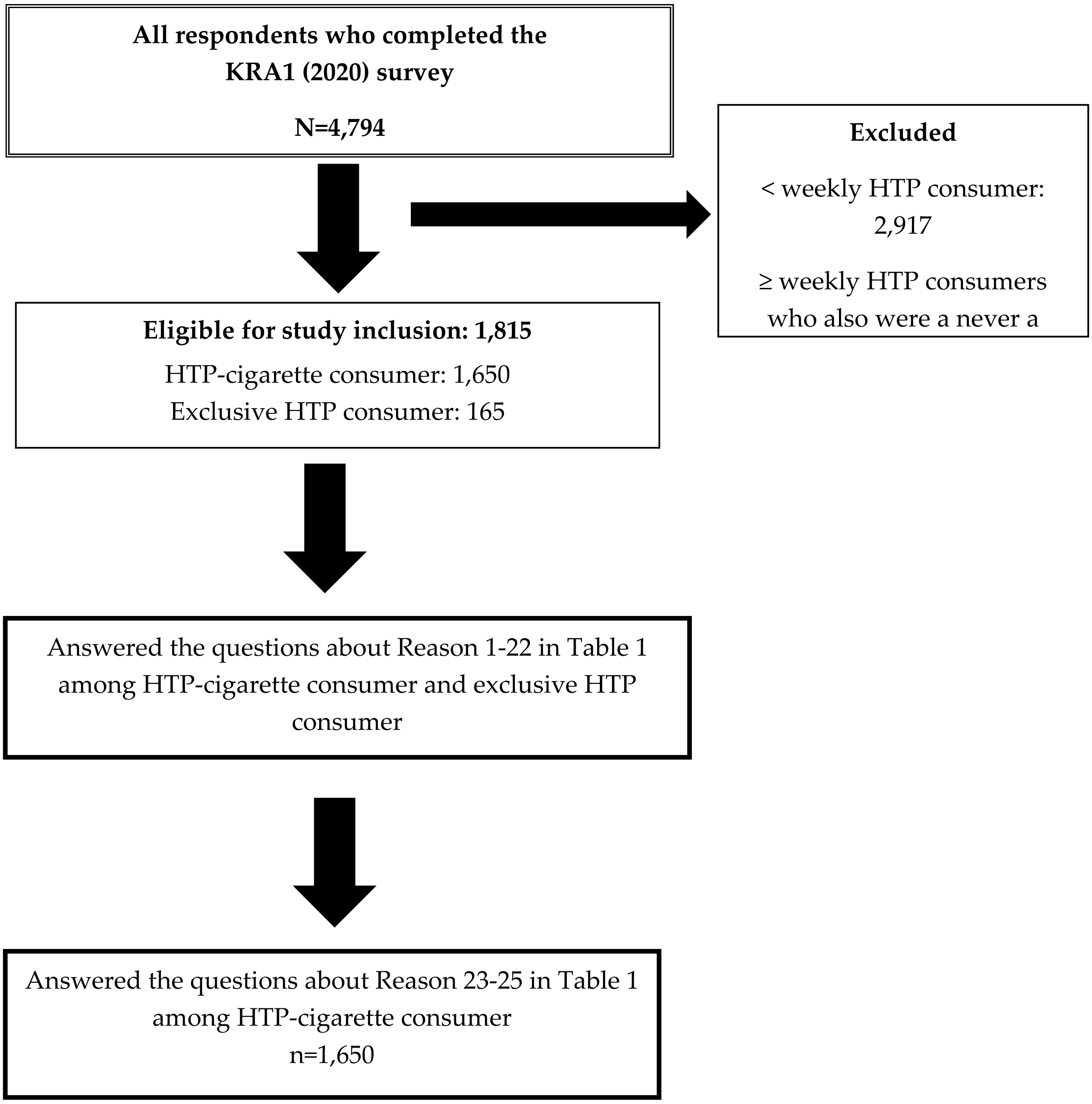

Supplementary Table S1. Themes and reasons for initiating and regularly using HTPs among dual HTP-cigarette and triple HTP-NVP-cigarette consumers in order of prevalence.

Theme/Reason	HTP-Cigarette Consumer	Dual HTP-Cigarette User	Triple User	Difference (*p*-Value)
	Weighted Percentage (95% Confidence Interval)			
Personal benefits: Less smelly than ordinary cigarettes	68.8 (66.0–72.3)	69.7 (65.6-73.5)	67.0 (60.4-73.1)	n.s
Initiation: Curiosity	60.8 (56.9–63.6)	61.7 (57.4-65.8)	58.7 (52.0-65.1)	n.s.
Harm reduction: Less harmful to own health	47.6 (44.3–51.1)	45.8 (41.5-50.3)	51.6 (45.0-58.0)	n.s
Personal benefits: Stress reduction	46.3 (43.4–50.3)	46.5 (42.1-51.0)	46.6 (40.1-46.6)	n.s.
Initiation: Family or friends use HTPs	47.5 (43.9–50.7)	47.2 (42.8-51.6)	49.8 (43.2-56.3)	n.s.
Personal benefits: Enjoyment	42.4 (39.8–46.6)	41.5 (37.3-45.9)	45.8 (39.3-52.3)	n.s.
Social consideration: More acceptable to others	40.6 (37.3–44.1)	41.3 (36.9-45.7)	38.7 (32.7-45.0)	n.s.
Harm reduction: Less harmful to others	40.6 (38.3–45.1)	?	?	n.s.
**Personal benefits: Taste good**	**39.7 (36.3–43.0)**	**37.7 (33.4-42.1)**	**46.4 (40.0-53.0)**	**0.029**
**Initiation: The heating/charging device is attractive**	**36.4 (33.0–39.5)**	**33.2 (29.3-37.5)**	**44.4 (38.0-50.9)**	**0.004**
**Initiation: Like the HTP technology**	**35.4 (32.3–38.8)**	**31.1 (27.1-35.5)**	**48.1 (41.6-54.7)**	**0.001**
**Convenience: Use in places where smoking cigarettes is banned**	**32.8 (29.6–36.0)**	**29.5 (25.6-33.7)**	**42.6 (36.3-49.3)**	**n.s.**
Personal benefits: Makes socializing easier	30.5 (27.7–34.0)	29.4 (25.5-33.6)	33.0 (27.3-39.2)	n.s.
**Product attractiveness: Attractive packaging**	**26.6 (23.4–29.3)**	**22.2 (18.7-26.0)**	**38.1 (32.1-44.4)**	**0.001**
Initiation: Offered by someone	25.8 (23.0–28.9)	23.8 (20.3-27.7)	30.5 (25.1-36.4)	n.s.
**Personal benefits: Something to occupy time with**	**25.5 (23.0–28.8)**	**20.4 (17.2-24.1)**	**38.9 (32.7-45.5)**	**0.001**
**Initiation: People in the media or other public figures use HTPs**	**22.3 (19.0–24.7)**	**16.8 (13.6-20.6)**	**36.2 (30.1-42.7)**	**0.001**
**Personal benefits: Save money**	**20.0 (17.2–22.5)**	**16.2 (13.1-19.7)**	**31.8 (26.3-37.9)**	**n.s.**
**Initiation: Experts like doctors and scientists use HTPs**	**19.1 (16.2–21.7)**	**16.4 (13.2-20.3)**	**29.0 (23.8-35.0)**	**0.001**
**Personal benefits: Control appetite and/or weight**	**18.5 (15.9–21.3)**	**?**	**?**	**0.001**
**Initiation: Health professionals advise switching to HTPs**	**17.4 (14.9–20.0)**	**13.7 (10.9-17.1)**	**27.3 (21.9-33.4)**	**0.001**

**Personal benefits: Look cool**	**16.0 (13.6–18.5)**	**13.2 (10.4-16.7)**	**23.3 (18.5-29.0)**	**0.001**
Intention**:** Cut down on the number of cigarettes I smoke	38.2 (34.9–41.7)	35.7 (31.5-40.0)	43.5 (37.1-50.1)	?
Intention: Using HTPs might help me quit smoking cigarettes	35.3 (32.0–38.8)	33.1 (29.0-37.5)	39.2 (33.0-45.8)	?
Intention**:** Replacing some cigarettes with HTPs to continue smoking	25.3 (22.3–28.5)	22.6 (19.0-26.6)	31.8 (26.0-38.1)	?

Bolded cells are those where the difference between dual HTP-cigarette and triple consumers is statistically significant at the $p<0.05$ level; n.s.: not statistically significant;
